# *Sdhd* and *Sdhd/H19* Knockout Mice Do Not Develop Paraganglioma or Pheochromocytoma

**DOI:** 10.1371/journal.pone.0007987

**Published:** 2009-11-24

**Authors:** Jean-Pierre Bayley, Ivonne van Minderhout, Pancras C. W. Hogendoorn, Cees J. Cornelisse, Annemieke van der Wal, Frans A. Prins, Luc Teppema, Albert Dahan, Peter Devilee, Peter E. M. Taschner

**Affiliations:** 1 Department of Human Genetics, Leiden University Medical Center, Leiden, The Netherlands; 2 Department of Anaesthesiology, Leiden University Medical Center, Leiden, The Netherlands; 3 Department of Pathology, Leiden University Medical Center, Leiden, The Netherlands; Deutsches Krebsforschungszentrum, Germany

## Abstract

**Background:**

Mitochondrial succinate dehydrogenase (SDH) is a component of both the tricarboxylic acid cycle and the electron transport chain. Mutations of SDHD, the first protein of intermediary metabolism shown to be involved in tumorigenesis, lead to the human tumors paraganglioma (PGL) and pheochromocytoma (PC). *SDHD* is remarkable in showing an ‘imprinted’ tumor suppressor phenotype. Mutations of *SDHD* show a very high penetrance in man and we postulated that knockout of *Sdhd* would lead to the development of PGL/PC, probably in aged mice.

**Methodology/Principal Findings:**

We generated a conventional knockout of *Sdhd* in the mouse, removing the entire third exon. We also crossed this mouse with a knockout of *H19*, a postulated imprinted modifier gene of *Sdhd* tumorigenesis, to evaluate if loss of these genes together would lead to the initiation or enhancement of tumor development. Homozygous knockout of *Sdhd* results in embryonic lethality. No paraganglioma or other tumor development was seen in *Sdhd* KO mice followed for their entire lifespan, in sharp contrast to the highly penetrant phenotype in humans. Heterozygous *Sdhd* KO mice did not show hyperplasia of paraganglioma-related tissues such as the carotid body or of the adrenal medulla, or any genotype-related pathology, with similar body and organ weights to wildtype mice. A cohort of *Sdhd*/*H19* KO mice developed several cases of profound cardiac hypertrophy, but showed no evidence of PGL/PC.

**Conclusions:**

Knockout of *Sdhd* in the mouse does not result in a disease phenotype. *H19* may not be an initiator of PGL/PC tumorigenesis.

## Introduction

Succinate dehydrogenase, subunit D (SDHD) is one of four proteins that together make up the mitochondrial tricarboxylic acid cycle enzyme, succinate dehydrogenase (SDH). In addition SDH plays an important role as the complex II component of the electron transport chain, ultimately leading to the generation of ATP by oxidative phosphorylation. Combining these roles places SDH at the center of two essential energy producing processes of the cell.

The identification of *SDHD* (chromosome 11q23) as a tumor suppressor gene revealed, for the first time, the involvement of both a mitochondrial protein and a protein of intermediary metabolism in tumorigenesis [1]. Mutations of *SDHD* lead to head and neck paragangliomas (HN-PGL), mainly benign tumors of the carotid body and other parasympathetically innervated paraganglia, but may also lead to tumors of the adrenal medulla (pheochromocytoma) and the sympathetically innervated paraganglia (extra-adrenal paraganglioma), some developing into aggressive metastatic cancers. Subsequently, two other subunits of SDH, *SDHC* (chromosome 1q21) [2], and *SDHB* (chromosome 1p36) [3] were implicated in paragangliomas.

A striking aspect of the natural history of *SDHD*-linked paraganglioma is the parent-of-origin inheritance of tumor susceptibility [4]. In contrast to paraganglioma in *SDHB* and *SDHC*-linked families, both located on chromosome 1, in *SDHD*-linked families and in the recently described SDH5 (*SDHAF2*) family [5], only a mutation inherited via the paternal line results in tumorigenesis. This strongly suggests the involvement of an imprinted locus in paragangliomas. No evidence exists to support the idea that these genes, both on chromosome 11, show monoallelic expression [1], [6]. The presence of the main cluster of imprinted human genes on the same chromosome, at 11p15.5, suggests a maternally expressed, imprinted gene as a compelling candidate for a modifier of tumor development. Loss of this gene, in addition to the maternal *SDHD* allele, may lead to the initiation of tumorigenesis. Loss of (maternal) chromosome 11 has been repeatedly demonstrated [6]–[8], counterintuitive if the maternal *SDHD* allele is imprinted and thus non-functional. This mechanism will result in a tumor that retains only the mutated paternal *SDHD* allele and entirely lacks active copies of all maternally expressed imprinted genes. Several genes on chromosome 11 are known to be exclusively maternally expressed including *CDKN1C*, *KCNQ1*, *KCNQ1DN*, *SLC22A18*, *PHLDA2*, *OSBPL5*, and *H19*. A well-described gene in the chromosome 11p15.5 region is *H19*, which has both a genetic and functional interaction with the paternally expressed insulin-like growth factor 2 (*IGF2*) gene. *H19* knockout mice are viable and display an overgrowth phenotype [9] and H19 has recently been shown to be a tumor modifier [10].

Here we report an *Sdhd* knockout mouse, lacking the entire third exon of *Sdhd*, which codes for the bulk of the active protein. This knockout mouse has been studied as a putative model for paraganglioma or pheochromocytoma. Tumor cohorts on two distinct inbred backgrounds were followed for their full life span, and analyzed in relation to *Sdhd*-related tumorigenesis, general pathology, and subtle hyperplasia of paraganglioma associated tissue.

To test the hypothesis that *H19* is the imprinted modifier gene, we crossed an existing *H19* knockout mouse line, Δ13, entirely lacking the *H19* gene and 10kb of the 5′ flanking region [9], with *Sdhd* knockout mice to assess effects on tumorigenesis. These mice were followed in an independent cohort for up to 29 months and monitored for signs of tumorigenesis.

## Results

### Generation of *Sdhd* Knockout Mice

**Figure 1:**
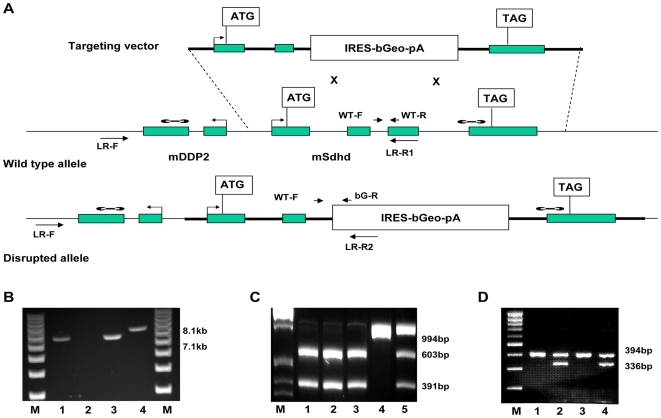
Generation and analysis of *Sdhd*-deficient mice. **A.** Schematic diagram of the strategy used to target the mouse *Sdhd* locus. The structure of the endogenous murine *Sdhd* gene (wildtype allele -wt) is shown in the middle with the targeting vector above and the disrupted allele below. Genomic DNA is represented by narrow horizontal lines with exons (shaded boxes), orientation of transcription (arrows), translation initiation and stop codons indicated; Genomic sequences flanking the betaGeo selection-reporter cassette (open box) in the targeting vector are represented by broad lines. Primers for genotyping (small arrows) and RT-PCR (arrowheads) are indicated below and above their target sequences, respectively. The dumbbells indicate the location of Southern blot probes used. **B.** Long-range PCR analysis of *Sdhd* gene targeting. The 7.1 kb normal allele amplified by primers LR-F and LR-R1 is present in the wildtype (lane 1) and heterozygous *Sdhd* knockout mouse (lane 3), whereas the 8.1 kb betaGeo targeted *Sdhd* allele amplified by primers LR-F and LR-R2 is present in the heterozygous *Sdhd* knockout mouse (lane 4) and not in the wildtype (lane 2) M = 10kb ladder marker. **C.** RT-PCR analysis of targeted gene expression. The C57BL/6J wt *Sdhd* allele is represented by 603bp and 391bp bands, and a 129P2/Ola wt allele, the 994bp band. Lanes 1 & 2; F1 pups from a C57BL/6J wt×129P2/Ola *Sdhd*+/− cross show only a single C57BL/6J wildtype *Sdhd* allele. Lanes 3, 4 & 5 = controls. Lane 3: C57BL/6J wt mouse. Lane 4: 129P2/Ola wt mouse. Lane 5: 129P2/Ola wt×C57BL/6J wt F1 mouse. The absence of a 129P2/Ola wildtype allele in the F1 offspring of the 129P2/Ola *Sdhd*+/−×C57BL/6Jwt cross demonstrates that they carry the *Sdhd*/betaGeo targeted allele, indicating correct targeting of the 129P2/Ola *Sdhd* locus. M = 100bp marker. **D.** Routine *Sdhd* genotyping of pups. M = marker, lanes 1 & 3; wt mice, primers WT-F & WT-R. Lanes 2 & 4; heterozygote mice, primers WT-F & bG-R.

**Figure 2:**
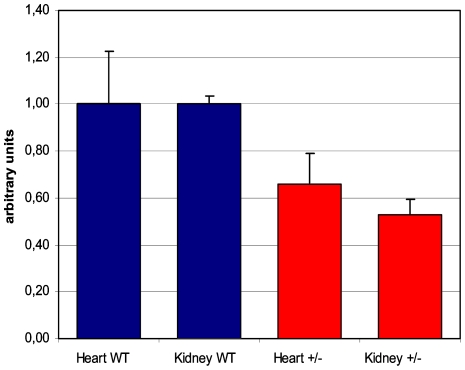
*Sdhd* expression in heart and kidney of one *Sdhd* ^+/+^ and two *Sdhd* ^+/−^ mice. Quantitative RT-PCR results were normalized to wildtype levels and expressed in arbitrary units. Error bars indicate 95% C.I.

An *Sdhd* targeting construct was designed in which the major coding exon of *Sdhd*, exon 3, was deleted and replaced with the betaGeo selection-reporter cassette (Figure 1A). The DY380 recombination competent E. coli strain was used in construct preparation, allowing direct recombination of sub-cloned fragments to generate the final targeting construct. Chimeric founder mice were crossed to wildtype female 129P2/Ola and C57BL/6J mice and germline transmission confirmed by long-range PCR and RT-PCR (Figure 1, B and C). The origin of wildtype *Sdhd* transcripts can be assessed by exon 3 to exon 4 RT-PCR and PvuII restriction analysis. Digestion with PvuII discriminates 129P2/Ola and C57BL/6J alleles. The absence of a 129P2/Ola wildtype allele in the F1 offspring of the 129P2/Ola Sdhd+/−×C57BL/6Jwt cross confirms correct targeting of the 129P2/Ola *Sdhd* locus at the RNA level (Figure 1, C). Routine genotyping was carried out using PCR analysis (Figure 1, D). Quantitative RT-PCR analysis of *Sdhd* expression in several tissues of *Sdhd*+/− mice showed that expression is reduced by approximately 50% (Figure 2).

### Heterozygous and Homozygous Gene Knockout of *Sdhd*

The loss of one allele and thus transcriptional potential of a gene with such a central role in intermediary metabolism as *Sdhd* may be significantly deleterious. Approximately equal numbers of wildtype and heterozygous *Sdhd* mice were obtained, indicating that these mice are fully viable (Table 1). Evaluation of the gross body weights of *Sdhd*+/− relative to wildtype littermates revealed no significant differences, either in males, females or in mice at a range of ages. Specific tissues, especially those normally showing high levels of SDH expression, such as heart or kidney, may show structural abnormalities or exhibit specific limitations in growth. Examination of these tissues (4 wt vs 4 healthy *Sdhd*+/− mice) revealed no differences. Complete lack of SDH/Complex II activity is unlikely to be compatible with life; therefore viability of homozygotes was examined. The expected Mendelian genotype ratios of 1∶2∶1 were not seen (Table 1). The absence of *Sdhd*−/− homozygotes among live offspring indicates that complete loss of *Sdhd* results in embryonic lethality.

**Table 1:** Sex and genotype ratios of *Sdhd* KO mice.

129P2/Ola F1*				
	Male	Female	+/+	+/−
n =	106	82	85	93
Ratio	1.3∶1.0		0.9∶1.0	

C57BL/6J F1				
	Male	Female	+/+	+/−
n =	139	121	138	122
Ratio	1.1∶1.0		1.1∶1.0	

129P2/Ola +/−×+/−			
	+/+	+/−	−/−
n =	52	101	0
Ratio	1.0∶2.0∶0.0		

* not all mice could not genotyped.

Sex and genotype ratios of the offspring of +/− mice backcrossed with wildtype mice of the 129P2/Ola and C57BL/6J genetic backgrounds are normal. Absence of live homozygous offspring from the crossing of heterozygous mice (129P2/Ola +/−**×**+/−) indicates that homozygous loss of *Sdhd* is not compatible with life.

Embryos isolated at 10.5 days onwards (n = 27) were developmentally normal and the genotype was found to be heterozygous. We conclude that lethality occurs at an earlier stage, in concordance with the findings of Piruat *et al* [11].

### Deletion of *Sdhd* Does Not Result in Tumorigenesis

**Figure 3:**
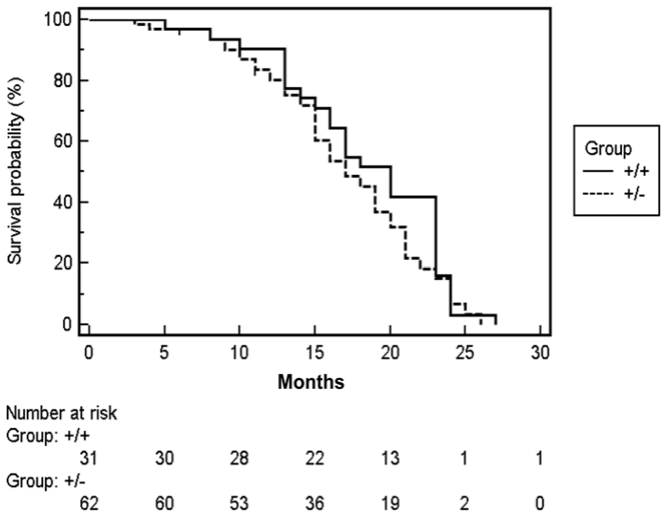
Kaplan-Meier survival data for the *Sdhd*+/− and *Sdhd*+/+ littermate cohort. Survival is shown as time to sacrifice (n = 93), determined by moribund state. The difference in survival is not significant (P = ns).

*SDHD* is a potent human tumor suppressor gene, heterozygous missense and nonsense mutations together with loss of heterozygosity (LOH), resulting in tumorigenesis [1]. A cohort was established to follow tumor development; including 93 mice with a homogenous 129P2/Ola background (62 *Sdhd*+/− mice and 31 wildtype), and a group of mice from a cross to C57BL/6J (n = 25). Because paraganglioma has a relatively late onset of >40 yrs in humans, these cohorts were followed for the entire lifespan of the mice for signs of tumorigenesis and general pathology. No gross indications of paraganglioma or pheochromocytoma were noted in any mice, at any age. A single case of approximately five-fold unilateral carotid body hyperplasia was noted in one *Sdhd*+/− mouse at 26 months. The mean age at death of all mice was 19 months (range 4–26 months). Survival curves revealed no difference in survival between *Sdhd*+/− and wt mice (Figure 3). Differentiated survival curves for males and females also showed no differences based on genotype. Examination of gross tissues and histological sections from moribund mice revealed a range of pathologies, but none of the pathology noted occurred significantly more frequently in the *Sdhd*+/− mice. We conclude that no genotype-specific pathology occurred in this cohort.

### No Significant Quantitative Change in the Carotid Body or Adrenal Medulla

**Figure 4:**
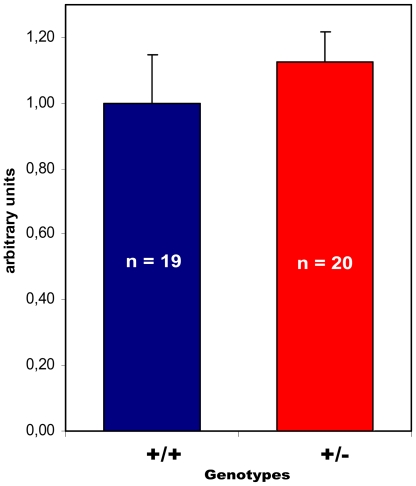
Relative size of *Sdhd*+/+ vs. *Sdhd*+/− mouse carotid bodies. The *Sdhd*+/+ to *Sdhd*+/− ratio was 1.0∶1.12 (P = ns). Error bars indicate 95% C.I.

Although no gross changes in the organs associated with paraganglioma (carotid body) or pheochromocytoma (adrenal medulla) were noted, the small size of these organs indicates a quantitative appraisal. Quantitative histological analysis of 39 carotid bodies (1682 serial HE sections) showed a 12% increase in size of the carotid body in *Sdhd*+/− mice relative to Sdhd+/+, though due to wide variation in the size of the carotid body, this difference was not significant (*P* = *0.19*) (Figure 4). The mouse displaying unilateral hyperplasia described above was excluded from the analysis. Representative sections of the adrenal medulla were also quantified in a similar manner and showed a similar but non-significant increase in total surface area.

**Figure 5:**
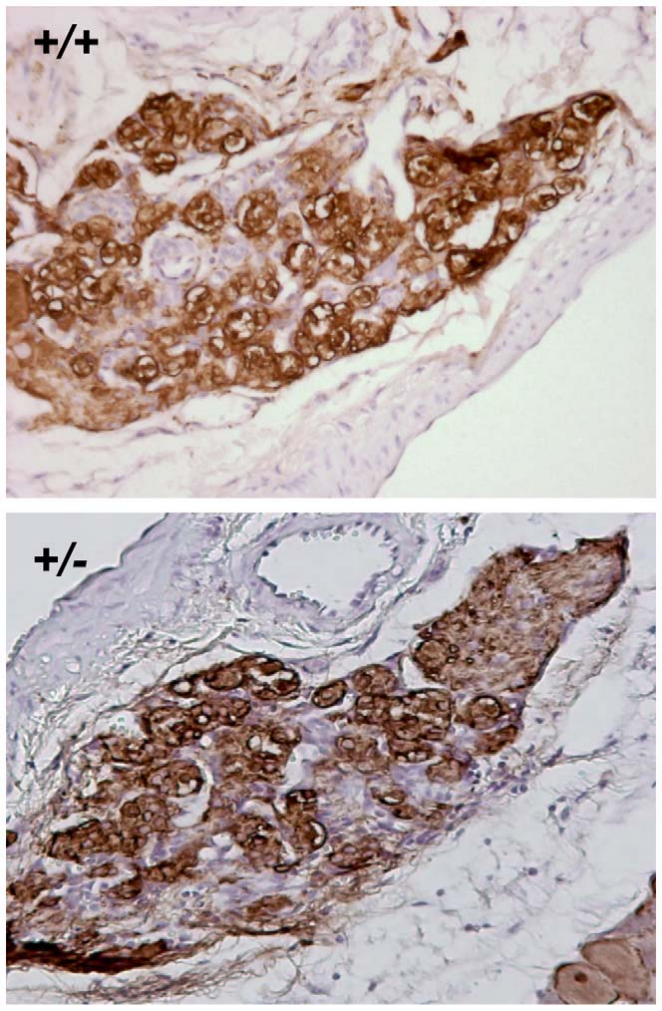
Tyrosine hydroxylase immunohistochemistry of the carotid body. Pictures are representative for completely serially sectioned carotid bodies from *Sdhd*+/+ and *Sdhd*+/− mice (n = 6 carotid bodies for *Sdhd*+/+ and 8 for *Sdhd*+/−). The ratio of *Sdhd*+/+ vs. *Sdhd*+/− TH staining is 1.0∶1.12 (P = 0.45).

Although the total surface area of carotid bodies of *Sdhd*+/− mice showed no significant increase in size, a change in the relative frequency of the cell types that constitute the carotid body would be significant in the context of neoplasia. To examine this possibility we quantified total surface area staining for chief cells using the specific marker, tyrosine hydroxylase (Figure 5). This analysis gave the same results as those for the HE staining described above, wt/het ratio 1∶1.12 (*P* = *0.45*), showing a trend to an increase in carotid body size in the heterozygotes but providing no statistical evidence for chief cell hyperplasia.

### *H19* as a Modifier of *Sdhd*

*SDHD*-linked paragangliomas in man show a striking ‘imprinted’ or parent-of-origin inheritance [4] and we have proposed a model including a maternally-expressed imprinted gene on chromosome 11 in *SDHD* tumorigenesis [6]. Because the orthologous imprinted locus in the mouse is located on chromosome 7 and *Sdhd* on chromosome 9, this mechanism is unlikely in the mouse. Therefore we crossed a knockout of a candidate imprinted modifier gene, the *H19* KO Δ13, to C57BL/6J *Sdhd*+/− mice. The resulting cohort consisted of 31 *Sdhd*+/−, *H19*+/− mice with the deletion inherited via the maternal line, and a similar numbers of controls, including *Sdhd*+/−; with *H19*+/− via the paternal line, and *Sdhd* wt; *H19*+/− via the maternal line. These mice were followed for their full lifespan of up to 29 months, but showed no pathology specific to the *Sdhd*+/− defect. Several cases of striking cardiac hypertrophy were noted but this was probably related to the genetic background or the defect in *H19*, also being seen in the *H19*+/− maternal line on an *Sdhd* wt background. No changes were noted in the carotid body or the adrenal medulla.

## Discussion

In man germline mutation of *SDHD* shows autosomal dominant inheritance with a penetrance of approximately 90% at age 70 [12]. In the *Sdhd* KO mice described here we saw no development of paraganglioma/pheochromocytoma at any age in two independent cohorts of mice followed for their entire lifespan. This divergence of genotype-phenotype correlation between man and mouse is far from unprecedented, and could be the result of any number of unknown physiological, biochemical or genetic mechanisms. What we do know is that there are clear differences in the chromosomal organization of genetic elements between the two species. While in man chromosome 11 harbours both *SDHD* (11q23) and the main locus for imprinted genes (11p15.5), these genes are on two separate chromosomes in the mouse (imprinted locus, chromosome 7 and *Sdhd*, chromosome 9).

The striking parent-of-origin inheritance pattern of *SDHD*-linked tumors in man strongly suggests the involvement of an imprinted locus in paragangliomas. All evidence indicates that *SDHD* itself is not imprinted [1], and we have postulated a role for an imprinted modifier gene on chromosome 11 in *SDHD*-related paraganglioma [6]. While *SDHD* and this modifier can be affected by whole chromosome loss in man, in the mouse loss of these genetic elements would require loss of two separate chromosomes, intrinsically less likely and perhaps incompatible with cell viability. The *H19*-*SDHD* double knockout included *H19* as the postulated *SDHD*-modifier, but did not lead to the development of paragangliomas even after 29 months. The modifier may not be *H19* but another maternally expressed gene on chromosome 11. Equally, because there was no tumor development in these mice, the true role of *H19* could not be properly evaluated [10]. While knockouts of other candidate genes exist, they generally result in pre- or perinatal lethality, precluding their use in the model postulated here. Inducible tissue-specific knockouts are not yet available. It is also worth noting that no case of spontaneous development of paraganglioma or pheochromocytoma has ever been reported in mice, perhaps suggesting an intrinsic resistance to the development of tumors of the paraganglia.

Knockout of orthologous human tumor suppressor genes in mice often results in the development of tumors of different tissue origin to that seen in man. Although a number of mice in our cohorts developed tumors of diverse types, there was no evidence that knockout of *Sdhd* was related to their genesis.

*Sdhd*+/− mice showed no notable differences in fertility, body mass, organ mass, or gross or histological morphology compared to wildtype littermates. The loss of one allele of a gene that is so essential to cellular metabolism might be expected to show some phenotypic expression, but can evidently be sufficiently compensated by transcription from the remaining allele. Any morphological changes of *Sdhd*+/− mice may be confined to a trend to a subtle increase in the size of the carotid body and adrenal medulla. This finding is concordant with a similar finding by Piruat *et al* [11], and suggests a subtle deficiency in *Sdhd* levels.

In conclusion, while the *Sdhd*+/− mouse described here is not a model for paraganglioma or pheochromocytoma, the aim to produce such a model remains valid, and should be further explored.

## Materials and Methods

### Ethics Statement

All mouse experiments were approved by the ethics committee for Animal Experiments of the University of Leiden and by the Commission Biotechnology in Animals of the Dutch Ministry of Agriculture. Following granting of ethical approval, all procedures were carried out in accordance with institutional policies.

### Mice

Wildtype mice were obtained from the Harlan UK, Bicester, England (129P2/OlaHsd) and Charles River Laboratories, France (C57BL/6JIco). H19 Δ13 KO mice [9] on a C57BL/6J background were a generous gift of Shirley Tilghman and John Levorse.

### Construction of the *Sdhd* betaGeo Targeting Vector and Generation of Knockout Mice

A 129/Sv mouse BAC genomic library was screened for Sdhd with primers for exon 4 (F 5′-TTGGACAAGTGGTTACCGACTAC-3′ and R 5′-ATGGCAACCGCTCTGCAGAT-3′) and primers for exon 1 (F 5′-GCAGGGCTCATCTTTCTC-3′ and R 5′-AGCTTTAAGAGAACCGCCAT-3′). Southern blotting, subcloning, and sequence analysis demonstrated that clone RPCI-21 560-8A contained the full *Sdhd* sequence. A targeting vector for the *Sdhd* locus was constructed on the basis of pU-Hachi, containing a SA – IRES - betaGeo cassette [13], (a generous gift of Dr. Kimi Araki). The betaGeo cassette includes a neomycin resistance gene and the E. coli betagalactosidase (lacZ) gene. The main coding exon of *Sdhd*, exon 3, was replaced by the betaGeo-IRES-cassette. As the 3′ homologous region, a 6.0-kb fragment EcoRI/BstZ17I fragment was subcloned, and the 5′ homologous region was a 3.1 kb BglII/BamHI fragment containing exon 4 of *Sdhd* subcloned 3′ of the betaGeo-IRES-cassette. The DY380 recombination competent E. coli strain (a generous gift of Dr. Shyam Sharan) was used in all following construction steps. After introduction of a zeomycin resistance cassette (from pZero) into the 3′ homologous region vector, the subcloned fragments were recombined in DY380 as described by Lee et al [14] and positive clones screened with PCR and Southern blotting.

129/Ola-derived embryonic stem (ES) cells [15] were transfected with the *Sdhd*-betaGeo targeting vector and screened by Southern blotting. In addition, X-gal staining for betagalactosidase activity gave an indication of correct targeting of the promoterless betaGeo-IRES-cassette to the *Sdhd* locus in ES cells. Two separately derived ES cell lines were used to generate the chimeric founders.

Chimeric mice were produced by injection of targeted ES cells into 3.5-day-old blastocysts using standard techniques. Germline transmission of KO alleles was analyzed by Southern blotting, long-range PCR and RT-PCR, and mice carrying the *Sdhd*-betaGeo allele were mated with C57BL/6J or 129P2/OlaHsd mice. Chimeras generated by ES cell targeting were crossed with 129P2/Ola females to transfer the 129P2/Ola-derived *Sdhd*-betaGeo construct on a homogenous 129P2/Ola background. Two separate chimeric lines were established. The data presented here are representative of one chimeric line, although both showed a similar phenotype. Mating of *Sdhd*+/− mice to the C57BL/6J mouse strain and selection for the *Sdhd* exon 3 deletion generated C57BL/6J-129P2/Ola *Sdhd*+/− mice. In contrast to all 129 strains, the C57BL/6J mouse strain carries a PvuII RFLP in exon 4 of *Sdhd*, which was used for genotyping. Except where indicated, all experiments were performed with *Sdhd*-betaGeo heterozygous mice and wildtype littermates obtained from matings of male heterozygous animals to wildtype female C57BL/6J or 129P2/OlaHsd mice. The 129P2/Ola cohort included 93 mice, and the 129P2/Ola *Sdhd*+/−×C57BL/6J F1 cohort 25 mice. Mice were housed in a 12-h light-dark cycle facility with free access to food and water.

### Southern Blotting, Long-Range PCR and Genotyping of Mice

Southern blotting was carried out using standard methods with 32P-labeled 5′ and 3′ probes (PCR product of mSdhdex2 primers, 5′-TCCGAAGCCGGGTGGTCAGA-3′ and 5′-GGTGGCTTGGTGACAGGTGA-3′) and (PCR product of mSdhdex4 primers 5′-TTGGACAAGTGGTTACCGACTAC-3′ and 5′-ATGGCAACCGCTCTGCAGAT-3′). DNA was digested with EcoRI or HindIII; EcoRI digestion yields a 7.1 kb fragment from the WT allele and a 6.2 kb allele from the *Sdhd*-betaGeo cassette with the mSdhdex4 probe, and mSdhdex2 a 3.6kb fragment from the WT allele and a 3.1 kb allele from the *Sdhd*-betaGeo cassette.

analysis of Sdhd gene targeting.

A 7.1 kb wildtype allele was amplified by primers LR-F and LR-R1 whereas an 8.1 kb betaGeo targeted *Sdhd* allele was amplified by primers LR-F and LR-R2. Inheritance of *Sdhd* wildtype and mutant alleles was monitored by duplex PCR analyses on genomic DNA obtained from tail biopsies with a trio of primers specific for wild-type forward WT-F and reverse wild-type WT-R or reverse mutant alleles bG-R. The three primers were used with the following amplification conditions: 95°C for 3 min, and 35 cycles of 95°C for 20 sec, 58°C for 20 sec, and 72°C for 20 sec, followed by 5 min at 72°C. Amplification products were resolved on a 1.8% agarose gel.

### RT-PCR Analysis and Real-Time PCR

Total cellular RNA was isolated from indicated adult mouse tissues using either RNA-Bee reagent (Tel-Test Inc, Texas, USA), or Trizol (Invitrogen BV, Leek, The Netherlands). 1ug of RNA was transcribed into cDNA with MMLV-RT (Invitrogen BV, Leek, The Netherlands) before being PCR amplified with primers specific for exon 3, or exon 4, of the Sdhd wildtype allele. PvuII digestion of the 994bp PCR product results in 603bp and 391bp fragments derived from the C57BL/6 allele and an undigested 129Ola fragment. The following amplification protocol was used: 95°C for 2 min, and 30 cycles of 95°C for 30 s, 58°C for 1 min, and 72°C for 1 min. Products were loaded onto a 1.2% agarose gel.

For quantitative real-time PCR (qPCR), total RNA was isolated from mouse heart and kidneys and cDNA generated as above. Experiments were performed using qPCR Corekits for SybrGreen or TAQman probes (Eurogentec, Seraing, Belgium). Cycle threshold (Ct) and starting quantities (SQ) were determined using the Biorad iCycler software (Biorad, Hercules, CA, USA). Ct and SQ values were normalised to the expression levels of four housekeeping genes, ActB, ActG, B2M, and Hprt using the geNorm program [16]. Statistical analysis (ANOVA) was carried out using SPSS 10.0 (SPSS Inc., Chicago, IL, USA). Mean *Sdhd* expression levels of two *Sdhd*+/− mice were compared to the mean levels in two wt littermates. The following primers were used: ActB (F- 5′-TTCTTTGCAGCTCCTTCGTTGC-3′, R- 5′-ACGACCAGC GCAGCGATATC-3′) and ActG (F- 5′-GCACTCTTCCAGCCTTCCTTCC-3′, R- 5′-GTAC CACCAGACAGCACTG TATTG-3′), B2m, (F- 5′-TTCAGTCGCGGTCGCTTCAG-3′, R- 5′-ATTTGAGGGGTTTTCTG GATAGCA-3′, and Hprt (F- 5′-AGTCCCAGCGTCG TGATTAGC-3′, R- 5′-GAGCAAGTCTT TCAGTCCTGTCC-3′). *Sdhd* primers were (F- 5′-CGAAAGCGACATGGCGGTTC-3′, R- 5′-GGTCCTGGAGAAATGCTGACAC-3′).

### Pathology and Histochemistry

On sacrifice of mice, a necropsy was performed and the weight and general condition of the animal noted. The condition and weight of organs was noted, and diseased tissue removed, fixed in 10% phosphate-buffered formalin, dehydrated, and embedded in paraffin. 10-um sections were stained with haematoxylin-eosin according to standard protocols. An investigator and a qualified pathologist examined the sections and a histopathological diagnosis was noted in case of abnormal findings.

For carotid body histochemistry and quantification, carotid bifurcations were removed bilaterally from wildtype and *Sdhd*-betaGeo heterozygous mice, fixed in 10% phosphate-buffered formalin, dehydrated, and embedded in paraffin. A series of carotid bodies (n = 39) were completely sectioned in 7-um serial sections (n = 40–60 per CB) and stained with haematoxylin-eosin according to standard protocols. Sections were photographed under a 10× objective and the total surface area of the carotid body quantified using Image J software (NIH, USA).

For quantitative immunohistochemistry, bilateral carotid bifurcations were removed from 6 wildtype and 6 *Sdhd*+/− mice, fixed in 10% phosphate-buffered formalin, dehydrated, and embedded in paraffin. The entire carotid body in 7-um serial sections was stained with an antibody specific for tyrosine hydroxylase (TH) (P40101-0. PelFreez, Arkansas, USA). Slides were incubated with the primary antibody (1∶500 dilution, o/n), followed by an anti-rabbit HPO secondary antibody for 30 min. Serial sections were photographed under a 10× objective and series quantified for total surface staining of TH in the carotid body using Image J software. Beta-galactosidase staining was on fresh tissue or frozen sections, fixed for 10 min in a 0.2% phosphate buffered glutaraldehyde solution (pH 7.4) containing 5 mM EGTA and 2 mM MgCl2. Beta-galactosidase activity was detected with 5-bromo-4-chloro-3-indolyl beta-D-galactopyranoside (X-gal) under standard conditions, followed optionally by counterstaining with Nuclear Fast Red.

## References

[1] Baysal BE, Ferrell RE, Willett-Brozick JE, Lawrence EC, Myssiorek D (2000). Mutations in SDHD, a mitochondrial complex II gene, in hereditary paraganglioma.. Science.

[2] Niemann S, Muller U (2000). Mutations in SDHC cause autosomal dominant paraganglioma, type 3.. Nat Genet.

[3] Astuti D, Latif F, Dallol A, Dahia PL, Douglas F (2001). Gene mutations in the succinate dehydrogenase subunit SDHB cause susceptibility to familial pheochromocytoma and to familial paraganglioma.. Am J Hum Genet.

[4] Van Der Mey AG, Maaswinkel-Mooy PD, Cornelisse CJ, Schmidt PH, van de Kamp JJ (1989). Genomic imprinting in hereditary glomus tumours: evidence for new genetic theory.. Lancet.

[5] Hao HX, Khalimonchuk O, Schraders M, Dephoure N, Bayley JP (2009). SDH5, a gene required for flavination of succinate dehydrogenase, is mutated in paraganglioma.. Science.

[6] Hensen EF, Jordanova ES, van Minderhout IJHM, Hogendoorn PCW, Taschner PEM (2004). Somatic loss of maternal chromosome 11 causes parent-of-origin-dependent inheritance in SDHD-linked paraganglioma and phaeochromocytoma families.. Oncogene.

[7] Margetts CDE, Astuti D, Gentle DC, Cooper WN, Cascon A (2005). Epigenetic analysis of HIC1, CASP8, FLIP, TSP1, DCR1, DCR2, DR4, DR5, KvDMR1, H19 and preferential 11p15.5 maternal-allele loss in von Hippel-Lindau and sporadic phaeochromocytomas.. Endocrine-Related Cancer.

[8] Riemann K, Sotlar K, Kupka S, Braun S, Zenner HP (2004). Chromosome 11 monosomy in conjunction with a mutated SDHD initiation codon in nonfamilial paraganglioma cases.. Cancer Genet Cytogenet.

[9] Leighton PA, Ingram RS, Eggenschwiler J, Efstratiadis A, Tilghman SM (1995). Disruption of Imprinting Caused by Deletion of the H19 Gene Region in Mice.. Nature.

[10] Yoshimizu T, Miroglio A, Ripoche MA, Gabory A, Vernucci M (2008). The H19 locus acts in vivo as a tumor suppressor.. Proc Natl Acad Sci U S A.

[11] Piruat JI, Pintado CO, Ortega-Saenz P, Roche M, Lopez-Barneo J (2004). The mitochondrial SDHD gene is required for early embryogenesis, and its partial deficiency results in persistent carotid body glomus cell activation with full responsiveness to hypoxia.. Mol Cell Biol.

[12] Benn DE, Gimenez-Roqueplo AP, Reilly JR, Bertherat J, Burgess J (2006). Clinical presentation and penetrance of pheochromocytoma/paraganglioma syndromes.. J Clin Endocrinol Metab.

[13] Araki K, Imaizumi T, Sekimoto T, Yoshinobu K, Yoshimuta J (1999). Exchangeable gene trap using the Cre/mutated lox system.. Cell Mol Biol (Noisy -le-grand).

[14] Lee EC, Yu DG, de Velasco JM, Tessarollo L, Swing DA (2001). A highly efficient Escherichia coli-based chromosome engineering system adapted for recombinogenic targeting and subcloning of BAC DNA.. Genomics.

[15] Hooper M, Hardy K, Handyside A, Hunter S, Monk M (1987). HPRT-deficient (Lesch-Nyhan) mouse embryos derived from germline colonization by cultured cells.. Nature.

[16] Vandesompele J, De Preter K, Pattyn F, Poppe B, Van Roy N (2002). Accurate normalization of real-time quantitative RT-PCR data by geometric averaging of multiple internal control genes.. Genome Biol.

